# Changes of body composition through an athlete-specific dietary guidance professional track and field athletes: a randomized controlled study

**DOI:** 10.1080/15502783.2026.2683613

**Published:** 2026-07-22

**Authors:** Ranil Jayawardena, Kalani Weerasinghe, Indu Nanayakkara, Terrence Madhujith, Andrew P. Hills, Nishan Sudheera Kalupahana

**Affiliations:** a Department of Physiology, Faculty of Medicine, University of Colombo, Colombo, Sri Lanka; b Department of Physiology, Faculty of Medicine, University of Peradeniya, Kandy, Sri Lanka; c School of Health Sciences, UTAS Health, University of Tasmania, Tasmania, Australia; d Health and Wellness Unit, Faculty of Medicine, University of Colombo, Colombo, Sri Lanka; e Department of Food Science and Technology, Faculty of Agriculture, University of Peradeniya, Kandy, Sri Lanka; f Department of Nutrition and Health, United Arab Emirates University, Al Ain, United Arab Emirates

**Keywords:** Body composition, personalized nutrition, track and field, Sri Lanka

## Abstract

**Background:**

Optimal body composition (BC) is essential for athletic success, influencing performance outcomes. Athlete-specific nutrition interventions are important for improving BC and enhancing performance, but evidence is limited, especially in track and field athletes. This study aimed to assess the effect of athlete-specific dietary guidance on BC in Sri Lankan track and field athletes.

**Methods:**

A 16-week trial was conducted with 30 athletes, 15 in each group: intervention (IG) and control (CG). The IG received athlete-specific dietary guidance through individual consultations at weeks 0, 4, and 8, along with weekly nutrition advice. BC was assessed using anthropometry, bioelectrical impedance analysis (BIA), and dual-energy X-ray absorptiometry (DEXA). Muscle strength, as a component of physical fitness, was assessed via handgrip strength (HGS).

**Results:**

Fourteen participants from each group completed the study. The IG demonstrated a significant decrease in fat mass percentage (FM) derived from BIA (Pre: 21.80 ± 7.0% vs. Post: 17.25 ± 5.8%; Change: −4.55 ± 1.2%; *p* = 0.003), while the CG demonstrated a no significant change (Pre: 18.87 ± 7.84% vs. Post: 19.60 ± 5.84%; Change: + 0.73 ± 3.0%; *p* = 0.475). The body fat percentage derived from DEXA also demonstrated significant reductions in the IG (Pre: 21.60 ± 7.1% vs. Post: 17.30 ± 6.0%; Change: −4.30 ± 1.1%; *p* = 0.005), and the CG exhibited a no significant change (Pre: 18.84 ± 3.9% vs. Post: 19.61 ± 6.5%; Change: + 0.77 ± 0.4%; *p* = 0.367). Fat-free mass (FFM) derived from DEXA increased in IG (Pre: 42.29 ± 6.0 kg vs. Post: 46.57 ± 6.4 kg; Change: +4.28 ± 0.4 kg; *p* < 0.001) when compared to CG (Pre: 39.13 ± 11.4 kg vs. Post: 38.05 ± 11.7 kg; Change: −1.08 ± 0.3 kg; *p* = 0.051). The IG showed significantly better HGS.

**Conclusions:**

Athlete-specific dietary guidance significantly improved BC and physical fitness among track and field athletes.

**Trial registration:**

This trial was registered in the Sri Lanka Clinical Trials Registry (SLCTR/2024/013) under Universal Trial Number (UTN) U1111-1304-8890 on 10 April 2024.

## 1. Introduction

In many sports, achieving an 'ideal' body composition (BC) is essential for competitive success, with specific characteristics depending on the demands of the sport [1]. According to the joint position stand by the American College of Sports Medicine, Dietitians of Canada, athletes should follow individualized nutrition strategies that align with their training demands, sport-specific needs, and health status to support peak performance and recovery [2]. These strategies include appropriate energy availability, macronutrient distribution (especially carbohydrate and protein timing), hydration and, where appropriate, evidence-based supplementation [2]. The International Society of Sports Nutrition also emphasizes that well-planned nutrition interventions can enhance BC, increase muscular strength, improve endurance capacity, and reduce injury risk when integrated with sport-specific training [3]. Dietary patterns that emphasize adequate protein, evenly distributed intake across meals, strategic carbohydrate timing, and the use of performance-supporting supplements such as creatine, caffeine, and *β*-alanine have shown consistent benefits across different athletic populations, including improvements in body composition, strength, endurance, and overall performance [4]. Therefore, understanding the relationship between BC and sports performance across various sports disciplines is crucial for enhancing athletes' performance parameters.

Ideal BC in athletes refers to an optimal balance of fat mass and fat-free mass that supports sport-specific performance, training adaptations, and overall health. Importantly, the optimal BC varies according to the athletic discipline and event demands. For example, endurance runners benefit from lower overall body fat and sufficient lean mass to improve running efficiency and endurance, whereas sprinters and jumpers require higher lower-body muscle mass and lean mass to support explosive power and speed, with moderate fat levels to optimize force production [5].

Reference values for total and regional BC, as well as anthropometric measures, have been established across sports and sexes, allowing sports professionals to evaluate whether observed changes in an athlete’s BC reflect favourable adaptations toward discipline-specific performance goals. Ideal total body fat percentages for male athletes generally range from approximately 6% to 20%, with endurance and aesthetic sports favouring the lower end of this range (around 6-12%) and strength or power sports tending toward the higher end (around 12−20%) [5]. For female athletes, ideal total body fat typically ranges from about 14% to 28%, with endurance and aesthetic sports at the lower end (approximately 14–20%) and strength or power sports at the higher end (around 20-28%) [5]. These ranges provide practical reference points for monitoring athlete adaptations and ensuring that fat mass and lean mass proportions align with performance and health goals specific to each athletic discipline.

Additionally, a lower body fat percentage can contribute to a higher power-to-weight ratio and enhanced performance in weightlifting and jumping tasks, which is crucial in sports where body weight must be propelled or moved quickly [6].

While BC is often managed to support sport-specific goals, it is essential that such strategies are approached cautiously to protect athletes' overall health and well-being [7]. Dietary regimens, such as a very low-calorie diet (VLCD) are commonly used by athletes aiming for rapid weight loss for competition, particularly in weight-class-specific sports [8]. While VLCDs have been shown to effectively reduce body and fat mass and improve metabolic health, they may also pose potential risks, including a reduction in fat-free mass, particularly skeletal muscle, which can lead to adverse consequences such as Relative Energy Deficiency in Sport (RED-S) [9,10].

Research indicates that daily supplementation with 25 μg of vitamin D may significantly impact athletes' BC, resulting in a considerable reduction in body fat percentage compared to the non-supplement group (1.9% vs. 0.2%, *p* = 0.02) [11]. Another study involving Iranian athletes showed that weekly vitamin D supplementation at a dose of 50,000 IU improves athletic performance in Iranian athletes, as evidenced by significant within-group improvements in leg press strength (*p* = 0.034) and sprint performance (one-repetition maximum, *p* = 0.030) [12]. Furthermore, research has indicated that protein supplementation both before and after resistance exercise enhances post-exercise protein synthesis and net muscle protein during recovery, thereby influencing BC [13]. Whey protein hydrolysate, in particular, may have a more pronounced effect on muscle hypertrophy when used in conjunction with resistance exercise training [14]. Moreover, the combined intake of whey proteins, branched-chain amino acids (BCAA), or creatine is more effective at increasing post-exercise muscle protein synthesis compared to protein alone [15]. In contrast, a separate trial found that a 7-day supplementation of BCAA (5 g/day) did not enhance running performance during a marathon and was ineffective in preventing muscle power loss, muscle damage, or perceived muscle pain during the race [16]. Another study found minimal benefit from glutamine supplementation for lean mass retention in athletes. Results reported that both the glutamine-supplemented and placebo groups experienced significant losses in body mass, lean body mass, and fat mass, with no significant differences between the groups following 12 days [17]. A randomized controlled trial supplementing with extra virgin olive oil demonstrated that fat loss was 80% greater compared to the control group (CG) (mean ± SE: −2.4 ± 0.3 kg vs. −1.3 ± 0.4 kg, *P* = 0.037), resulting in improved body composition [18].

Previous intervention studies have demonstrated that targeted nutrition support can lead to meaningful reductions in body fat and improvements in lean mass, outcomes that are highly desirable for athletic performance. For instance, in elite athletes, nutritional intervention over 8–12 weeks produced significant gains in lean body mass compared to ad libitum intake [19]. Similarly, a study involving adolescent and adult athletes receiving dietary counselling reported increased lean body mass and decreased fat mass following the intervention [20]. For athletes undergoing weight loss, raising protein intake (to ~2.3 g/kg) helped preserve fat-free mass compared with lower protein diets [21]. Moreover, Ryan et al. conducted a 12-week nutrition intervention in 25 high-school male athletes, reporting significant reductions in body fat percentage and fat mass, along with increases in fat-free mass (*p* < 0.05) [22]. Taken together, these findings suggest that the most desirable BC changes in athletes namely, reductions in body fat percentage and increases in fat-free mass can be achieved through tailored nutrition strategies.

Besides, sports nutrition interventions play a crucial role in contributing to shifts in body composition in line with sport-specific performance needs. Most studies have focused on altering either energy intake, increasing protein intake, or providing sports supplements to healthy individuals and observing their impact on BC. However, in real-life situations, the cumulative effect of total energy intake and energy composition from different macronutrients, especially protein and micronutrient supplementation, along with the use of ergogenic supplements in specific sports, are responsible for changes in BC. Hence, in our study, we aim to assess the changes in BC among professional track and field athletes through an evidence-based, culturally accepted, athlete-specific sports nutrition intervention. South Asians, on average, have a higher total body fat percentage for a given body mass index (BMI) compared to White populations [23]. This population demonstrates a greater accumulation of intermuscular and visceral fat, coupled with lower lean body mass than many other ethnic groups [24]. Despite South Asia constituting approximately one-quarter of the global population, the region's athletic performance on the Olympic stage remains suboptimal [25]. While athletic performance is influenced by multifactorial determinants, unhealthy BC may be a significant contributing factor for this population [26]. Hence, in our study, we specifically aimed to evaluate changes in BC, particularly reductions in body fat percentage and improvements in fat-free mass among professional track and field athletes through an evidence-based, culturally accepted, athlete-specific sports nutrition intervention.

## 2. Methods

The CONSORT 2010 statement guidelines regarding randomized trials (www.consort-statement.org) were followed for this randomized controlled clinical trial (RCT) [27].

### 2.1. Trial design

**Figure 1.**
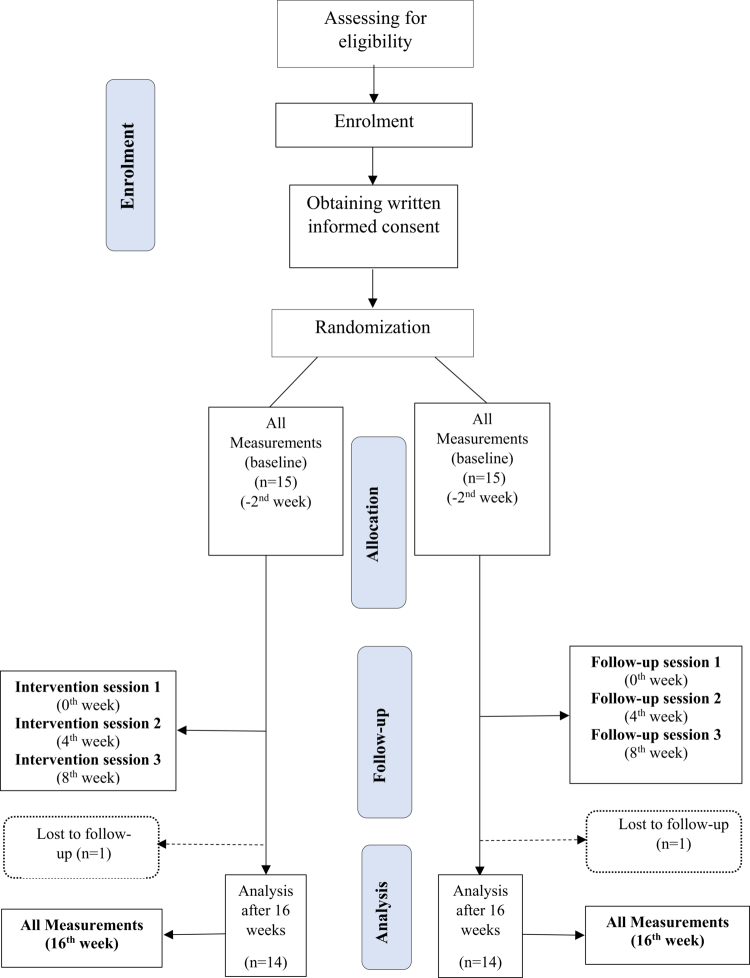
CONSORT diagram.

This study was designed as a parallel-group, randomized controlled clinical trial conducted over 16 weeks at the Department of Physiology, Faculty of Medicine, University of Colombo, Sri Lanka. Written informed consent was obtained from all participants upon recruitment, in accordance with the Helsinki Declaration. Ethical approval was granted by the Institutional Ethical Review Committee at the Faculty of Medicine, University of Peradeniya, Sri Lanka (2023/EC/71). This trial is registered with the Sri Lanka Clinical Trials Registry (SLCTR/2024/013), with a Universal Trial Number (UTN): U1111-1304-8890. The CONSORT diagram (Figure 1) illustrates the flow of this RCT.

### 2.2. Participants

Eligible participants included elite or highly trained track and field athletes of both genders. Potential subjects were recruited through contacts from coaches, fellow athletes, and open advertisements in social media groups. Participants were screened two weeks prior to the intervention and required to provide verbal and written informed consent. Inclusion criteria were national-level athletes aged 18 and above, male and female, with a personal WhatsApp number, and full-time athletes willing to participate in the 16-week nutrition intervention. Athletes with current sports-related injuries, those following a prescribed diet or taking sports supplements, and individuals who relied primarily on ready-to-eat fast food or ultra-processed food for much of their daily intake were excluded, to ensure that dietary guidance could be effectively implemented. A total of 30 participants were recruited and randomly allocated to the IG and CG, with 15 participants in each group. The formula used for sample size calculation has been published elsewhere [28].

### 2.3. Randomization and allocation

Eligible participants were randomized into the IG or CG in a 1:1 ratio using a computer-generated random sequence [29]. Allocation concealment was ensured by using sealed opaque envelopes prepared in advance by an independent researcher not involved in the study. These envelopes were opened sequentially at the time of enrolment, ensuring allocation was not known until assignment.

### 2.4. Intervention

Evidence-based, culturally accepted, and athlete-specific dietary guidance was provided to the intervention group (IG) after all relevant baseline measurements and investigations. The primary focus of the intervention was optimizing dietary intake to meet energy, macronutrient, and micronutrient needs tailored to each athlete’s sport-specific training and performance goals. The key elements of the athlete-specific diet plan are as follows:

− Providing sufficient energy according to training loads and activity levels.
− Ensuring protein intake of 1.2–1.6 g/kg body weight, distributed across four meals (0.3–0.4 g/kg per meal) [30].
− Encouraging extra virgin olive oil (EVOO) and other Mono-Unsaturated Fatty Acid (MUFA) sources [18] while discouraging Saturated Fatty Acids (SFA), especially coconut oil.
− Prescribing multivitamins and calcium for everyone and prescribe vitamin D and iron when deficiencies are diagnosed biochemically.
− For those who cannot achieve the required amount of energy and protein from dietary sources, prescribing calorie-containing supplements, and whey protein.
− Recommending ergogenic supplements (e.g. creatine) for some athletes according to current guidelines [31].

Athletes in the IG were provided evidence-based nutrition guidance. . Specifically, creatine supplementation was offered only to athletes who participated in power- or strength-based track and field events (e.g. sprinters, jumpers, throwers) and who were not already taking supplements. The decision to provide creatine was based on individual training demands, sport-specific performance goals, and established evidence-based guidelines. Supplement recommendations were made in alignment with the IOC consensus statement on dietary supplements and the high-performance athlete [31], ensuring adherence to internationally recognized best practices.

Additionally, basic dietary advice was provided, which included consuming high-glycaemic index (GI) carbohydrate-rich foods before training; protein- and carbohydrate-rich foods as post-training meals; maintaining proper hydration; practising good sleep hygiene; consuming a variety of fruits and vegetables; and avoiding energy-dense, nutrient-poor ultra-processed foods such as sugary snacks, fried fast foods, and sugar-sweetened beverages. Athlete-specific dietary guidance was delivered through 15–30-minute individual in-person consultations at weeks 0, 4, and 8, conducted by the principal investigator (RJ). In-person dietary consultations were conducted at weeks 0, 4, and 8 to provide initial guidance, reinforce adherence, and review progress during the first half of the intervention. These sessions introduced and individualized the dietary guidance according to each athlete’s BC, training status, and personal preferences. During the latter 8 weeks, no scheduled in-person consultations were conducted, allowing athletes to independently apply the guidance while receiving ongoing remote support. Throughout the 16-week intervention, participants had access to continuous support via weekly remote check-ins and a WhatsApp-based hotline for clarification and practical dietary advice, including adaptations when prescribed foods were unaffordable or unavailable. This structure aligned with ethics committee recommendations, emphasizing structured in-person support initially, followed by online monitoring and self-management. During the latter 8 weeks, no further in-person sessions were conducted to allow athletes to independently apply the nutrition guidance while maintaining weekly remote support via WhatsApp. This approach was designed to encourage self-management and evaluate the sustainability of dietary adherence under real-life conditions. In the second half of the intervention, weekly sports nutrition education materials adapted from national sports nutrition guidelines [32] were shared via WhatsApp (weeks 10-16), in addition to personalized feedback and clarification through WhatsApp calls or chats. All athletes attended the three scheduled in-person consultations. Moreover, a 24/7 hotline was maintained via WhatsApp to provide timely clarification and additional nutrition advice. Although no further in-person sessions were conducted, athletes were able to contact the research team for follow-up guidance. Suggestions were also provided when athletes were unable to afford or access the prescribed food items. The extent of additional advice varied, as some athletes sought frequent support while others required minimal input, likely reflecting individual differences in comprehension, nutritional literacy, and confidence in implementing the guidance.

Participants in the CG were instructed to maintain their usual dietary habits throughout the 16-week period and were not required to follow any structured weight-loss or performance-focused diet. No specific dietary advice was provided to the CG during the intervention phase. At baseline and post-intervention, all participants completed a seven-day food diary to document their habitual eating practices. At the end of the study, athletes in the CG were provided with basic nutrition advice.

### 2.5. Compliance monitoring and dietary assessment

Athlete adherence to the dietary guidance was monitored through multiple approaches. First, participants received weekly check-ins via WhatsApp messages, where they reported adherence, raised questions, and received clarifications. Second, athletes completed seven-day food diaries both prior to the intervention and at the end of the 16-week intervention. These diaries recorded the type and portion of all foods consumed, which were analyzed to assess compliance with the prescribed nutrition plan. The total intake over seven days was averaged and reported as pre- and post-intervention values, allowing us to evaluate dietary changes and adherence to the intervention. In addition, research assistants maintained a 24-hour, 7-day hotline facility to address any queries from athletes in real time. Where athletes found it difficult to afford or access specific prescribed foods, practical and context-appropriate alternatives were suggested to support adherence while maintaining the intended nutritional balance.

### 2.6. Outcome measures

I.
**Anthropometry**

Anthropometric measurements such as body weight, height, mid-upper arm circumference (MUAC), waist circumference and triceps skinfold thickness (TSFT) were measured using calibrated equipment by trained research assistants, following international recommendations. Details are published elsewhere [28].

II.
**Body c** **omposition measurements**

Bio-electrical impedance analysis (BIA) was performed to measure resistance, reactance, phase angle, and impedance values (Body Stat, Douglas, Isle of Man, UK). Fat-free mass (FFM) was calculated using sex-specific equations developed by Rush et al. for Asian Indians [33], incorporating height, total body weight, and BIA variables such as reactance and resistance. Fat mass (FM) was determined by subtracting FFM from total body weight, and fat mass percentage (FM%) was calculated using FM and total body weight.

Sex-Specific Equations for FFM:

For Men:

FFM = 0.382 × $\frac{H^{2}}{R}$ + 0.167W + 0.320 H−36.382

For Women:

FFM = 0.456 × $\frac{H^{2}}{R}$ + 0.127 W + 0.0746 X + 5.959

where:

FFM = Fat-Free Mass (kg)

H = Height (cm)

R = Resistance

W = Weight (kg)

X = Reactance

To ensure accuracy and reproducibility of BIA assessments, participants were provided with standardized pre-test instructions, including fasting overnight (≥8 hours), avoiding strenuous physical activity and alcohol consumption for at least 24 hours, and voiding their bladder within 30 minutes prior to measurement. All BIA measurements were performed under similar conditions in the morning.

Additionally, equations based on anthropometric measurements, including MUAC and TSFT, were utilized to estimate the upper arm muscle area (UAMA) and upper arm fat area (UAFA) [34].

The formulas applied were:

UAMA (cm²) = [(MUAC − (π × TSFT)) ²]/(4 × *π*)

UAFA (cm²) = [(MUAC²)/(4 × *π*)]—UAMA

TSFT was selected as it is a reliable and validated indicator of subcutaneous fat in South Asian populations, providing a practical measure of regional fat distribution [35] that complements BIA and Dual-energy X-ray Absorptiometry (DEXA) assessments. Moreover, taking multiple skinfold measurements would increase participant burden; hence, we limited the assessment to TSFT.

DEXA was employed to measure bone mineral density, bone mineral content (BMC), fat mass, total body fat percentage (TBF%), fat-free mass, and the combined measurement of fat-free tissue mass and lean mass, which includes both fat-free tissue and BMC in the appendicular regions of the body. DEXA scans (QDR 4500 A, Waltham, USA) were conducted at an accredited radiography unit (Nawaloka Hospital, Colombo, Sri Lanka).

All BC measurements (BIA, anthropometry, and DEXA) were obtained at baseline (week 0) and post-intervention (week 16) to assess changes resulting from the intervention.

III.
**Hand grip strength (HGS)**

HGS was selected as it is a simple, reliable, and validated indicator of overall muscular strength and functional capacity and has been shown to correlate with athletic performance in various sports populations, including track and field athletes [36]. HGS is also frequently used in sports science research as a practical tool to assess muscular strength and functional capacity. This was measured in kilograms using a calibrated hydraulic hand dynamometer (Model SH5001^®^; SAEHAN Corporation, YangdeokDong, Masan, South Korea), according to the guidelines by the American Society of Hand Therapists [37]. Each participant was seated during the assessment, and measurements were taken three times for each hand. The highest value obtained was used for analysis.

IV.
**Physical activity and training**

Total energy expenditure was estimated using a seven-day activity diary, dividing each day into 96 periods of 15 minutes. Participants completed the diaries once at baseline (0^th^ week) and once post-intervention (16^th^ week), with detailed instructions and demonstrations provided to ensure accurate recording. Participants were instructed on how to complete the diary, and the primary activity undertaken during each reporting period was recorded. These records were processed to calculate physical activity levels using the physical activity ratio value of each category and the predicted BMR formula (BMR = 0.074 × kg BW + 2.754 MJ/d for men, and BMR = 0.056 × kg BW + 2.898 MJ/d for women) [38].

V.
**Total calorie calculation**

Participants were asked to record all foods and beverages consumed, including portion sizes, both before the intervention (two-week lead-in) and after the intervention (end of week 16), using seven-day food diaries in a booklet provided with written and verbal instructions. The total intake over seven days was averaged to generate pre- and post-intervention values. The energy content of each food item was calculated using standard energy values, while macronutrient and micronutrient intake were analyzed with Nutri-Survey Software modified for Sri Lankan foods. In addition, the principal investigator (RJ) collected verbal dietary histories from all athletes at baseline (week 0) and endpoint (week 16) to cross-validate the diary data and provide supplementary estimates of total caloric intake. This approach allowed for accurate estimation of total daily calorie intake and assessment of diet quality.

### 2.7. Statistical methods

Descriptive statistics were used to summarize participant characteristics. Independent sample t-tests and paired t-tests were employed to compare baseline and end-of-study characteristics between groups for normally distributed continuous variables and to evaluate within-group changes. Effect sizes were calculated using Cohen’s d to quantify the magnitude of between- and within-group differences and were interpreted as small (0.2), medium (0.5), and large (0.8). All analyses were performed using SPSS version 23 (SPSS Inc., Chicago, IL, USA).

## 3. Results

Among the 30 participants who initially enrolled, 14 from each group (IG and CG) completed this 16-week trial (Figure 1). The results for the 28 participants who completed the research are presented using the per-protocol analysis approach.

The mean age of the IG was 22.1 ± 2.5 years, with a gender distribution of 8 males (57%) and 6 females (43%). The CG had a mean age of 21.9 ± 3.9 years, with 9 males (64%) and 5 females (36%). The baseline demographic and anthropometric characteristics of the participants are summarized in Table 1. The average sports experience was 7.15 ± 3.60 years for the IG and 5.92 ± 3.73 years for the CG (*p* = 0.394). Regarding performance levels, 36% of participants in both groups were classified as elite athletes, while 64% were categorized as highly trained athletes. Participants were engaged in various sports disciplines, and a detailed distribution is provided in Table 1.

**Table 1.** Baseline demographic and anthropometric values of study participants.

Variable	IG (*n* = 14) Mean ± SD	CG (*n* = 14) Mean ± SD	**p*-*value
Age (y)	22.1 ± 2.5	21.9 ± 3.9	0.260
Gender			1.000
Male	8 (57%)	9 (64%)	
Female	6 (43%)	5 (36%)	
Height (cm)	1.68 ± 0.7	1.70 ± 1.0	0.223
Weight (kg)	55.08 ± 9.9	52.96 ± 9.1	0.546
BMI (kgm^−2^)	19.37 ± 1.80	18.64 ± 1.73	0.293
Sports experience (y): *n* (%)	7.15 ± 3.60	5.92 ± 3.73	0.394
Level of performance: *n* (%)			0.394
Elite	5 (36%)	5 (36%)	
Highly trained	9 (64%)	9 (64%)	
Main sport event: *n* (%)			1.000
Sprinting	1 (7%)	1 (7%)	
Middle-distance running	7 (50%)	10 (71%)	
Long-distance running	3 (21%)	2 (14%)	
Jumping	2 (14%)	1 (8%)	
Throwing	1 (7%)	0 (0%)	
TBF%	21.60 ± 7.1	18.84 ± 3.9	0.120
MUAC (cm)	25.00 ± 2.8	23.96 ± 2.7	0.342
WC (cm)	70.41 ± 8.1	67.94 ± 5.8	0.363
TSFT (mm)	8.83 ± 6.0	6.45 ± 2.5	0.185
BIA Impedance	600.07 ± 67.7	612.36 ± 76.0	0.655
BIA Resistance	595.21 ± 67.6	607.79 ± 75.8	0.647
BIA Reactance	74.7 ± 8.1	73.47 ± 9.4	0.715
BIA PA	7.20 ± 0.6	6.91 ± 0.6	0.253
FM (kg)	11.64 ± 6.6	8.63 ± 3.3	0.144
Lean Mass (kg)	42.29 ± 6.0	39.13 ± 11.4	0.370
BMC (kg/m²)	2246.0 ± 275.1	2060.7 ± 698.5	0.364
Lean and BMC indices (kg/m²)	15.83 ± 1.4	13.6 ± 1.3	0.327
Appendicular mass (kg/m²)	7.59 ± 0.9	8.11 ± 0.9	0.832
FFM (kg): BIA	42.15 ± 7.0	40.20 ± 7.6	0.621
FM%: BIA	21.80 ± 7.0	18.87 ± 7.8	0.700
UAMA (cm^2^)	4.54 ± 4.9	7.52 ± 8.9	0.054
UAFA (cm^2^)	43.76 ± 10.7	47.01 ± 10.9	0.055

AL and BMC indices: Appendicular Lean and Bone Mineral Content indices, BIA Impedance: Bioelectrical Impedance Analysis Impedance, BIA PA: Bioelectrical Impedance Analysis Phase Angle, BIA Reactance: Bioelectrical Impedance Analysis Reactance, BIA Resistance: Bioelectrical Impedance Analysis Resistance, BMC (kg): Bone Mineral Content (kilograms), BMI: Body Mass Index, CG: Control Group, Fat mass (kg): Fat Mass (kilograms), FFM BIA: Fat-Free Mass (measured by using BIA), FM% BIA: Fat Mass Percentage (measured by using BIA), IG: Intervention Group.

The IG demonstrated significant increases in energy and protein intake over the 16-week period, whereas no significant changes were observed in the CG; full dietary intake data are provided in Supplementary Table 1.

At baseline, the TBF% was 21.60 ± 7.1 in the IG and 18.84 ± 3.9 in the CG (*p* = 0.120). The IG recorded mean MUAC, WC, and TSFT values of 25.00 ± 2.8 cm, 70.41 ± 8.1 cm, and 8.83 ± 6.0 mm, respectively, while the CG had corresponding values of 23.96 ± 2.7 cm, 67.94 ± 5.8 cm, and 6.45 ± 2.5 mm. The BIA parameters, including resistance, reactance, and PA values, did not show significant differences between the groups. The FFM derived from BIA was 42.15 ± 7.0 kg in the IG and 40.20 ± 7.6 kg in the CG (*p* = 0.621), and FM was 21.80 ± 7.0 kg in the IG and 18.87 ± 7.8 kg in the CG (*p* = 0.700).

Following the intervention, significant improvements were observed in the IG for key anthropometric measures (Table 2). MUAC showed a significant improvement in the IG (Pre: 25.00 ± 2.8 cm vs. Post: 21.52 ± 1.8 cm; Change: −3.48 ± 1.0 cm; *p* < 0.001; d = 1.42, large effect), indicating a reduction in muscle circumference. In contrast, no significant changes were observed in the CG (Pre: 23.96 ± 2.7 cm vs. Post: 24.01 ± 2.6 cm; Change: +0.04 ± 0.1 cm; *p* = 0.772). Waist circumference (WC) also exhibited a significant decrease in the IG (Pre: 70.41 ± 8.1 cm vs. Post: 67.03 ± 6.5 cm; Change: −1.01 ± 1.5 cm; *p* = 0.013; d = 0.59, medium effect), while the CG experienced a no significant change (Pre: 67.94 ± 5.8 cm vs. Post: 69.30 ± 5.7 cm; Change: + 1.36 ± 0.1 cm; *p* = 0.210).

**Table 2.** Comparison of changes in anthropometric and body composition parameters between the IG and.

Variable	IG (*n* = 14) Mean ± SD					CG (*n* = 14) Mean ± SD					*p*-value for IG vs CG		
	Pre	Post	Change	*p*-value	Cohen’s d	Pre	Post	Change	*p*-value	Cohen’s d	Pre	Post	Change
Weight (kg)	55.08 ± 9.9	56.85 ± 9.5	+ 1.77 ± 0.4	0.180	0.18	52.96 ± 9.1	53.15 ± 9.5	+ 0.19 ± 0.3	0.780	0.02	0.546	0.504	0.564
MUAC (cm)	25.00 ± 2.8	21.52 ± 1.8	−3.48 ± 1.0	<0.001	1.42	23.96 ± 2.7	24.01 ± 2.6	+ 0.04 ± 0.1	0.772	0.02	0.342	0.007	0.012
WC (cm)	70.41 ± 8.1	67.03 ± 6.5	−1.01 ± 1.5	0.013	0.59	67.94 ± 5.8	69.30 ± 5.7	+ 1.36 ± 0.1	0.210	0.25	0.363	0.339	0.030
TSFT (mm)	8.83 ± 6.0	7.85 ± 5.5	−0.98 ± 0.4	0.071	0.23	6.45 ± 2.5	6.38 ± 2.8	+ 0.07 ± 0.3	0.753	0.02	0.185	0.387	0.054
BIA Impedance	600.07 ± 67.7	619.5 ± 70.2	+ 19.43 ± 2.5	0.053	0.28	612.36 ± 76.0	618.71 ± 78.0	-0.172 ± 1.9	0.503	0.00	0.655	0.978	<0.001
BIA Resistance	595.21 ± 67.6	614.36 ± 70.5	+ 19.15 ± 2.9	0.055	0.27	607.79 ± 75.8	614.21 ± 77.5	-0.1321 ± 1.7	0.490	0.00	0.647	0.996	<0.001
BIA Reactance	74.7 ± 8.1	79.22 ± 7.4	+ 4.52 ± 0.7	0.031	0.60	73.47 ± 9.4	75.19 ± 10.9	+ 1.72 ± 1.5	0.390	0.18	0.715	0.266	<0.001
BIA PA	7.20 ± 0.6	7.37 ± 0.8	+ 0.16 ± 0.2	0.343	0.22	6.91 ± 0.6	7.02 ± 0.7	+ 0.12 ± 0.1	0.239	0.17	0.253	0.245	0.560
FM (kg)	11.64 ± 6.6	10.27 ± 5.2	−1.37 ± 1.4	0.025	0.33	8.63 ± 3.3	9.71 ± 9.2	+ 1.08 ± 5.9	0.051	0.18	0.144	0.149	<0.001
Fat-free Mass (kg)	42.29 ± 6.0	46.57 ± 6.4	+ 4.28 ± 0.4	< 0.001	0.68	39.13 ± 11.4	38.05 ± 11.7	-1.08 ± 0.3	0.051	0.19	0.370	0.025	<0.001
BMC (kg/m²)	2246.0 ± 275.1	2253.1 ± 274.4	+ 7.1 ± 0.7	0.593	0.03	2060.7 ± 698.5	2050.3 ± 689.0	-10.4 ± 9.5	0.159	0.01	0.364	0.365	<0.001
Lean and BMC indices (kg/m²)	15.83 ± 1.4	16.05 ± 1.5	+ 0.22 ± 0.1	0.440	0.15	13.6 ± 1.3	13.00 ± 1.2	-0.60 ± 0.1	0.334	0.46	0.327	0.328	<0.001
Appendicular mass (kg/m²)	7.59 ± 0.9	7.23 ± 0.8	+ 0.36 ± 0.1	0.079	0.43	8.11 ± 0.9	7.30 ± 0.8	-0.81 ± 0.1	0.271	0.90	0.832	0.050	0.002
FFM (kg): BIA	42.15 ± 7.0	46.43 ± 6.1	+ 4.28 ± 0.9	<0.001	0.63	40.20 ± 7.6	38.77 ± 6.1	+ 1.43 ± 1.5	0.124	0.19	0.621	0.023	<0.001
FM%: BIA	21.80 ± 7.0	17.25 ± 5.8	+ 4.55 ± 1.2	0.003	0.64	18.87 ± 7.84	19.60 ± 5.84	+ 0.73 ± 3.0	0.475	0.06	0.700	0.614	<0.001
UAMA (cm^2^)	4.54 ± 4.9	5.12 ± 5.2	+ 0.58 ± 0.3	0.567	0.12	7.52 ± 8.9	7.11 ± 7.2	-0.41 ± 1.7	0.662	0.05	0.054	0.018	0.002
UAFA (cm^2^)	43.76 ± 10.7	31.29 ± 4.6	12.47 ± 6.1	<0.001	2.04	37.60 ± 5.9	36.41 ± 6.3	1.19 ± 0.4	0.077	0.09	0.058	0.742	<0.001

AL and BMC indices: Appendicular Lean and Bone Mineral Content indices, BIA Impedance: Bioelectrical Impedance Analysis Impedance, BIA PA: Bioelectrical Impedance Analysis Phase Angle, BIA Reactance: Bioelectrical Impedance Analysis Reactance, BIA Resistance: Bioelectrical Impedance Analysis Resistance, BMC (kg): Bone Mineral Content (kilograms), CG: Control Group, Fat mass (kg): Fat Mass (kilograms), FFM BIA: Fat-Free Mass (measured by using BIA), FM% BIA: Fat Mass Percentage (measured by using BIA), IG: Intervention Group.

*p-*value is considered significant at < 0.05.

**Figure 2.**
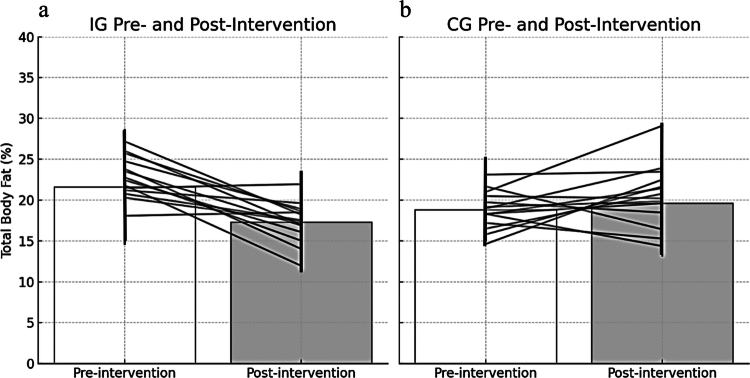
a. Change in total body fat% (DEXA) from pre- to post-intervention in the intervention group. b**.** Change in total body fat% (DEXA) from pre- to post-intervention in the control group.

In terms of body composition, TBF% significantly decreased in the IG (−4.55 ± 1.2%; *p* = 0.003; d = 0.64, medium-to-large effect). The CG showed no significant change in TBF% (Pre: 18.84 ± 3.9 vs. Post: 19.61 ± 6.5; Change: + 0.77 ± 0.4; *p* = 0.367) (Figures 2a and b).

**Figure 3.**
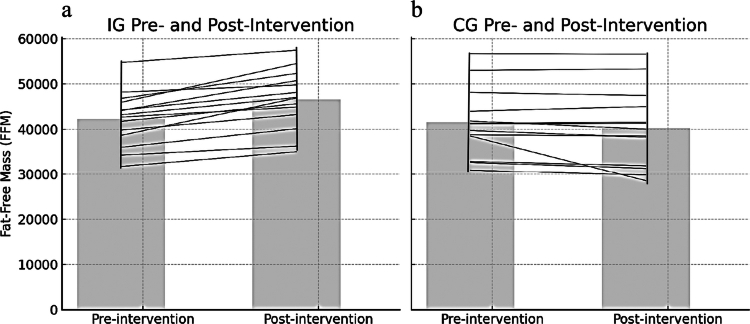
a. Change in fat-free mass (kg) derived from DEXA scan from pre- to post-intervention in the intervention group. b. Change in fat-free mass (kg) derived from DEXA scan from pre- to post-intervention in the control group.

According to DEXA results, fat-free mass significantly increased in the IG (Pre: 42.29 kg ± 6.0 vs. Post: 46.57 kg ± 6.4; Change: + 4.28 kg ± 0.4; *p* < 0.001; d = 0.68, medium-to-large effect) but decreased in the CG (Pre: 39.13 kg ± 11.4 vs. Post: 38.05 kg ± 11.7; Change: −1.08 kg ± 0.3; *p* = 0.051; for change *p* < 0.001). FM significantly reduced in the IG (Pre: 11.64 kg ± 6.6 vs. Post: 10.27 kg ± 5.2; Change: −1.37 kg ± 1.4; *p* = 0.025; d = 0.33, small-to-medium effect), while the CG experienced a no significant change (Pre: 8.63 kg ± 3.3 vs. Post: 9.71 kg ± 9.2; Change: + 1.08 kg ± 5.9; *p* = 0.051; change *p* < 0.001). UAFA in the IG showed a notable reduction (Pre: 43.76 cm^2^ ± 10.7 vs. Post: 31.29 cm^2^ ± 4.6; Change: -12.47 cm^2^ ± 6.1; *p* < 0.001; d = 2.04, very large effect), contrasting with the CG, which remained unchanged (Pre: 37.60 cm^2^ ± 5.9 vs. Post: 36.41 cm^2^ ± 6.3; Change: −1.19 cm^2^ ± 0.4; *p* = 0.077; change *p* < 0.001) (Figure 3a and b).

**Figure 4.**
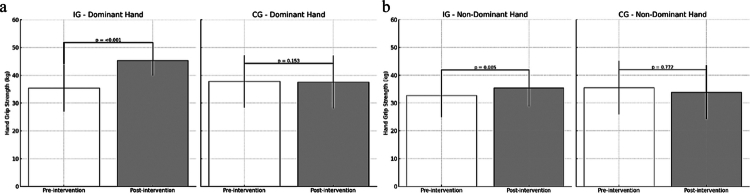
a. Comparison of changes in mean hand grip strength values of the dominant hand between the intervention and control groups. b. Comparison of changes in mean hand grip strength values of the non-dominant hand between the intervention and control groups.

HGS demonstrated significant improvements in the IG following the intervention. For the dominant hand, mean HGS increased from 35.38 ± 8.46 kg at baseline to 45.30 ± 5.44 kg post-intervention (Change: + 9.92 ± 3.02 kg; *p* < 0.001; d = 1.17, large effect). For the non-dominant hand, mean HGS increased from 32.70 ± 7.73 kg to 35.39 ± 6.67 kg (Change: + 2.69 ± 1.06 kg; *p* = 0.005; d = 0.42, medium effect). In contrast, the control group (CG) showed no significant changes in either hand (Figures 4a and b).

## 4. Discussion

To our knowledge, this is the first study conducted among South Asian track and field athletes to improve body composition through an athlete-specific dietary intervention without altering training regimens. The present study demonstrated favourable changes in multiple body composition outcomes, including reductions in total body fat percentage and fat mass, alongside increases in fat-free mass, following a 16-week dietary guidance programme. These findings highlight the potential of evidence-based nutrition strategies to positively influence body composition parameters relevant to athletic performance when implemented safely.

Certain BC profiles, such as lower body fat and higher fat-free mass, have been associated with enhanced athletic performance in specific sports contexts [39]. Body fat percentage has been shown to influence sprint performance and explosive power, as measured by vertical jump tests [40]. An observational analytic study revealed a positive correlation (*p* = 0.001; R = 0.732) between body fat percentage and sprint speed, indicating that a higher body fat percentage is associated with slower sprint times among futsal athletes [40]. Additionally, a negative correlation (*p* = 0.001; R = -0.639) was found between body fat percentage and cardiorespiratory endurance, suggesting that greater body fat percentage is linked to lower endurance levels [40]. Additionally, studies have highlighted correlations between muscle mass percentage and attributes like agility or aerobic capacity in sports similar to futsal, such as handball, hockey, and volleyball [41–43]. Kooshaki et al. [44] further linked the anthropometric characteristics of female futsal players to key skills, including ball control, sprinting, passing, dribbling, and shooting. Their findings revealed a negative association where higher fat percentages adversely impact sporting performance [44]. Moreover, research indicates that an elevated body fat percentage may increase the likelihood of overuse injuries, such as patellar tendinopathy and rotator cuff tendinopathy, particularly in basketball players [45,46].

An exclusive focus on athletes’ perceived optimal body composition may cause athletes, coaches, and support staff to place undue emphasis on physical appearance and physique rather than holistic determinants of performance [47]. Societal ideals and expectations within the sport, influenced by coaches, teammates, parents, or the sport's culture, may pressure athletes to attain a specific ‘lean athletic look’ [48,49]. This pressure to reduce body mass or body fat without appropriate justification can result in body dissatisfaction and contribute to disordered eating and eating disorders [50]. Furthermore, the inappropriate setting of BC goals, particularly focusing on low body mass or body fat levels, can lead to low energy availability, potentially resulting in RED-S [51]. This condition can cause malnutrition, muscle mass loss, hormonal disturbances, growth and development disruption, and psychological impairments [52]. It is important to acknowledge that while reductions in body fat percentage and increases in fat-free mass are generally associated with improved performance, there are potential risks if such changes are pursued excessively. Very low body fat levels, particularly below 6-8% in males and 12-14% in females may compromise health and increase the risk of hormonal disturbances, impaired recovery, and reduced immunity [50,51]. Furthermore, athletes who attempt to modify body composition without adequate caloric intake may be at risk of disordered eating behaviours or clinical eating disorders. In our study, the intervention emphasized balanced dietary guidance tailored to Sri Lankan food patterns, rather than restrictive dieting. Both before and after the intervention, athletes were encouraged to maintain adequate caloric intake to support training and recovery, and no reports of restrictive or disordered eating behaviours were observed. Nevertheless, future studies should more directly monitor energy availability and eating behaviours to ensure that positive changes in body composition are not achieved at the expense of athlete health.

Empirical evidence indicates an optimal protein intake of 1.2-1.6g/kg/BW of protein, mostly in four divided meals, achieving 0.3-0.4 g/kg/BW per meal [30]. Research shows that athletes often have an uneven distribution of protein intake, with larger portions consumed during dinner [53]. Current recommendations suggest an intake of around 1.5 g/kg of body weight distributed evenly over 4-5 meals, especially post-training [54]. Additionally, whey protein supplementation has been shown to increase muscle mass and reduce fat mass, benefiting athletes [55]. Replacing saturated fatty acids (SFA), especially coconut oil, with EVOO is important for muscle rebuilding [18]. Creatine supplementation may also be advantageous, particularly for sprinters, jumpers, and throwers, by improving anaerobic work capacity and enhancing performances that require multiple surges in intensity or during final sprints [56]. Addressing vitamin and mineral deficiencies, such as ensuring adequate vitamin D levels, can significantly impact body composition, as vitamin D is crucial for the regulation of fat and muscle mass, contributing to improved BC outcomes. Together with BMI, vitamin D deficiency is independently related to increased regional subcutaneous fat tissue [57].

Our sample exhibited relatively higher TBF% for both males (Pre: 17.83 ± 2.6) and females (Pre: 24.32 ± 2.0) compared to the reference values reported for track and field athletes. The ideal TBF% ranges are 6–12% for males and 12–20% for females in this population [5]. However, following the intervention, a decrease in TBF% was observed, approaching ranges commonly reported for track and field athletes. While these changes may reflect adaptations that align with sport-specific demands, care must be taken not to overinterpret such changes as inherently beneficial without considering individual health and performance goals. By the end of the study, a directional reduction in body fat percentage was seen (Post: Males: 15.92 ± 3.5; Females: 22.39 ± 4.7) [58]. Additionally, increases in FFM and decreases in FM were observed, with minimal changes in overall body weight. In the present study, participants’ dietary intake was closely monitored using seven-day food diaries before and after the 16-week intervention, supplemented by verbal dietary histories to cross-validate the data. DEXA analysis confirmed significant increases in FFM, reflecting improvements in LBM following the intervention. These adaptations are consistent with the observed increase in HGS, suggesting that improvements in muscularity were functionally relevant. Despite this, no major changes in overall energy or macronutrient intake were detected, indicating that the intervention stimulus, rather than dietary alterations, likely drove these adaptations. Future studies could incorporate more precise compliance tracking and controlled dietary interventions to further explore the relationship between nutrient intake and changes in LBM.

Muscle mass showed improvement across both genders. No significant changes in bone mineral content were observed, which is likely attributable to the relatively short duration of the intervention. Minor changes observed in the control group may reflect natural variability, seasonal training effects, or behavioural changes associated with study participation (i.e. a Hawthorne effect). However, these changes were small, non-significant, and unlikely to represent systematic improvements attributable to structured dietary guidance.

A key strength of our study is that it demonstrates measurable changes in BC and physical function without modifying exercise routines. By avoiding any implication that changes in BC are universally beneficial, this study supports the use of individualized and culturally appropriate nutrition strategies to support athletic performance. The inclusion of culturally accepted dietary components enhances the feasibility and applicability of the intervention, particularly in a Sri Lankan context. Regular consultations (0, 4, 8 weeks) and continuous weekly advice through WhatsApp messaging provide consistent support for the athletes, facilitating adherence to the intervention and promoting long-term changes in dietary habits.

However, the study has several limitations. First, the sample size was relatively small, which may limit the generalizability of the findings. It is also important to consider that the athletes represented a range of track and field disciplines, including sprinters, jumpers, and middle-distance runners. Sport-specific physiological demands may partly explain the variability in responses to the intervention. For instance, sprinters and throwers often benefit from greater fat-free mass to optimize power output, whereas distance runners may prioritize a lower body fat percentage to improve running economy. Although subgroup analyses were not conducted due to the limited sample size, such differences may have influenced the degree of change observed in body composition. Future studies should explore sport-specific adaptations to nutritional interventions, ideally stratifying athletes by discipline to clarify whether responses vary according to the unique physiological demands of their event. Additionally, we included only HGS, but incorporating more sophisticated measures such as VO₂ max, 1-RM, sprint tests, and vertical jump tests could have provided a more detailed assessment of the intervention's impact on athletic physical functionality. Therefore, future studies should include a larger sample, extend the duration, and examine multiple variables.

## 5. Conclusions

In conclusion, this 16-week evidence-based sports nutrition intervention demonstrated significant improvements in BC and physical fitness for athletes in the IG compared to the CG, by reducing TBF%, WC, MUAC, and UAFA, as well as increasing FFM and HGS. These results suggest that athlete-specific nutrition interventions can positively impact BC and physical performance without modifying exercise routines among track and field athletes.

## Supplementary Material

Supplementary Material All_supplementary_materials.docx

## Data Availability

Not applicable.
