# Supplementary material for: Changes of body composition through an athlete-specific dietary guidance professional track and field athletes: a randomized controlled study
Source: J Int Soc Sports Nutr. 2026 Jul 22;23(1):2683613. doi: 10.1080/15502783.2026.2683613 (PMC13393057; doi:10.1080/15502783.2026.2683613)
Supplement: Supplementary Material — All_supplementary_materials.docx [file RSSN_A_2683613_SM5537.docx]

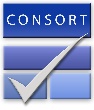
Supplementary material 1. CONSORT 2010 checklist of information to include when reporting a randomised trial

| Section/Topic | Item No | Checklist item | Reported on page No |
| --- | --- | --- | --- |
| Title and abstract | | | |
|  | 1a | Identification as a randomised trial in the title | 1 |
|  | 1b | Structured summary of trial design, methods, results, and conclusions (for specific guidance see CONSORT for abstracts) | 2 |
| Introduction | | | |
| Background and objectives | 2a | Scientific background and explanation of rationale | 3, 4 |
|  | 2b | Specific objectives or hypotheses | 5 |
| Methods | | | |
| Trial design | 3a | Description of trial design (such as parallel, factorial) including allocation ratio | 6 |
|  | 3b | Important changes to methods after trial commencement (such as eligibility criteria), with reasons | NA |
| Participants | 4a | Eligibility criteria for participants | 6 |
|  | 4b | Settings and locations where the data were collected | 6 |
| Interventions | 5 | The interventions for each group with sufficient details to allow replication, including how and when they were actually administered | 7 |
| Outcomes | 6a | Completely defined pre-specified primary and secondary outcome measures, including how and when they were assessed | 8 |
|  | 6b | Any changes to trial outcomes after the trial commenced, with reasons | NA |
| Sample size | 7a | How sample size was determined | 8 |
|  | 7b | When applicable, explanation of any interim analyses and stopping guidelines | NA |
| Randomisation: |  |  |  |
| Sequence generation | 8a | Method used to generate the random allocation sequence | 8 |
|  | 8b | Type of randomisation; details of any restriction (such as blocking and block size) | 8 |
| Allocation concealment mechanism | 9 | Mechanism used to implement the random allocation sequence (such as sequentially numbered containers), describing any steps taken to conceal the sequence until interventions were assigned | 8 |
| Implementation | 10 | Who generated the random allocation sequence, who enrolled participants, and who assigned participants to interventions | 8 |
| Blinding | 11a | If done, who was blinded after assignment to interventions (for example, participants, care providers, those assessing outcomes) and how | 8 |
|  | 11b | If relevant, description of the similarity of interventions | NA |
| Statistical methods | 12a | Statistical methods used to compare groups for primary and secondary outcomes | 8 |
|  | 12b | Methods for additional analyses, such as subgroup analyses and adjusted analyses | 8, 9 |
| Results | | | |
| Participant flow (a diagram is strongly recommended) | 13a | For each group, the numbers of participants who were randomly assigned, received intended treatment, and were analysed for the primary outcome | 10 |
|  | 13b | For each group, losses and exclusions after randomisation, together with reasons | 10 |
| Recruitment | 14a | Dates defining the periods of recruitment and follow-up | 10 |
|  | 14b | Why the trial ended or was stopped | NA |
| Baseline data | 15 | A table showing baseline demographic and clinical characteristics for each group | Table 1 |
| Numbers analysed | 16 | For each group, number of participants (denominator) included in each analysis and whether the analysis was by original assigned groups | 10 |
| Outcomes and estimation | 17a | For each primary and secondary outcome, results for each group, and the estimated effect size and its precision (such as 95% confidence interval) | 11 Table 2, Figure 1, 2, 3, 4a. 4b |
|  | 17b | For binary outcomes, presentation of both absolute and relative effect sizes is recommended | NA |
| Ancillary analyses | 18 | Results of any other analyses performed, including subgroup analyses and adjusted analyses, distinguishing pre-specified from exploratory | NA |
| Harms | 19 | All important harms or unintended effects in each group (for specific guidance see CONSORT for harms) | NA |
| Discussion | | | |
| Limitations | 20 | Trial limitations, addressing sources of potential bias, imprecision, and, if relevant, multiplicity of analyses | 13 |
| Generalisability | 21 | Generalisability (external validity, applicability) of the trial findings | 13 |
| Interpretation | 22 | Interpretation consistent with results, balancing benefits and harms, and considering other relevant evidence | 12 |
| Other information | | |  |
| Registration | 23 | Registration number and name of trial registry | 17, 18 |
| Protocol | 24 | Where the full trial protocol can be accessed, if available | 17, 18 |
| Funding | 25 | Sources of funding and other support (such as supply of drugs), role of funders | 17, 18 |

**Supplementary material 2.** Calculation of FFM using Bioelectrical Impedance Analysis

FFM was calculated using sex-specific equations developed by Rush and colleagues for Asian Indians [22], incorporating height, total body weight, and BIA variables such as reactance and resistance. Fat mass (FM) was derived by subtracting FFM from total body weight, and fat mass percentage (FM%) was calculated as the ratio of FM to total body weight.

*Sex-Specific Equations for FFM:*

**For men:**

FFM=0.382×$\frac{H^{2}}{R}$ +0.167W+0.320 H−36.382

**For women:**

FFM=0.456×$\frac{H^{2}}{R}$ +0.127 W+0.0746 X+5.959

Where:

FFM = Fat-Free Mass (kg)

H = Height (cm)

R = Resistance

W = Weight (kg)

X = Reactance

| Variable | IG (n=13)  Mean ± SD | | | | | CG (n=14)  Mean ± SD | | | | p-value for IG vs CG | | |
| --- | --- | --- | --- | --- | --- | --- | --- | --- | --- | --- | --- | --- |
|  | Pre | Post | Change | p-value | Pre | | Post | Change | p-value | Pre | Post | Change |
| Energy (kcal) | 2766.0±494.0 | 3972.9±934.4 | +1206.9 | 0.007 | 2733.7±635.5 | | 2676.5±319.2 | -57.2 | 0.739 | 0.884 | 0.047 | 0.004 |
| Carbohydrate (g) | 441.2±52.1 | 475.3±132.9 | +34.1 | 0.373 | 445.9±105.7 | | 444.9±87.9 | -1.0 | 0.976 | 0.883 | 0.097 | 0.240 |
| Carb % (%) | 62.62±7.2 | 61.44±6.6 | -1.18 | 0.132 | 63.57±6.0 | | 65.38±7.3 | +1.81 | 0.419 | 0.714 | 0.155 | 0.012 |
| Protein (g) | 95.56±30.2 | 116.81±41.8 | +21.25 | 0.003 | 94.54±32.8 | | 84.24±16.4 | -10.30 | 0.162 | 0.933 | 0.019 | 0.001 |
| Protein % (%) | 13.10±2.1 | 15.26±3.6 | +2.16 | 0.006 | 13.24±3.1 | | 12.48±2.7 | -0.76 | 0.079 | 0.898 | 0.036 | 0.005 |
| Fat % (%) | 24.36±5.3 | 20.10±4.0 | -4.26 | 0.477 | 23.19±4.9 | | 22.24±5.99 | -0.95 | 0.747 | 0.656 | 0.252 | <0.001 |
| DF (g) | 16.53±8.7 | 19.16±11.1 | +2.63 | 0.037 | 19.07±9.2 | | 12.21±6.8 | -6.86 | 0.638 | 0.563 | 0.663 | 0.027 |
| PUFA (g) | 9.75±3.6 | 19.42±4.5 | +9.67 | 0.002 | 9.68±5.1 | | 7.88±2.8 | -1.80 | 0.048 | 0.468 | 0.037 | <0.001 |

**Supplementary table 1.** Comparison of changes in dietary nutrients, vitamins and minerals between the IG and CG
